# Glymphatic dysfunction and neuroinflammation in FXTAS: evidence from DTI-ALPS and gene expression analysis

**DOI:** 10.3389/fnmol.2026.1856101

**Published:** 2026-05-28

**Authors:** Andrea Elias-Mas, Esther Granell Moreno, Cèlia Painous Martí, Marta Rubio-Roy, Jorge Aguado-Gracia, Emma Muñoz-Moreno, Alejandro Hinojosa, Iñigo Herrero, Maria Isabel Alvarez-Mora, Randi Hagerman, Jun Yi Wang, Idoia Zaro, Sofia González Ortiz, Jerzy Krupinski, Laia Rodriguez-Revenga

**Affiliations:** 1 Radiology Department, Hospital Universitari Mútua Terrassa, (HUMT), Terrassa, Spain; 2 Institute for Research and Innovation Parc Taulí (I3PT), Sabadell, Spain; 3 Genetics Doctorate Program, Universitat de Barcelona (UB), Barcelona, Spain; 4 Radiology Department, Hospital de Santa Creu i Sant Pau, Barcelona, Spain; 5 Unitat de Parkinson i altres Trastorns del Moviment, Servei de Neurologia, Hospital Clínic de Barcelona, FRCB-IDIBAPS, Institut de Neurosciències, CIBERNED, ISCIII, ERN-RND, UBNeuro, Barcelona, Spain; 6 Department of Neurology, Parc Taulí Hospital Universitari, Sabadell, Spain; 7 Department of Child and Adolescent Psychiatry and Psychology, Hospital Clinic of Barcelona, Barcelona, Spain; 8 Institut d’Investigacions Biomèdiques August Pi i Sunyer (IDIBAPS), Barcelona, Spain; 9 Biochemistry and Molecular Genetics Department, Hospital Clinic of Barcelona, Barcelona, Spain; 10 CIBER of Rare Diseases (CIBERER), Instituto de Salud Carlos III, Barcelona, Spain; 11 MIND Institute, University of California Davis, Sacramento, CA, United States; 12 Department of Pediatrics, University of California Davis, School of Medicine, Sacramento, CA, United States; 13 Department of Psychiatry and Behavioral Sciences, University of California Davis, School of Medicine, Sacramento, CA, United States; 14 Radiology Department, Hospital Clínic de Barcelona, Barcelona, Spain; 15 Department of Neurology, F.Ass. MútuaTerrassa, Barcelona, Spain; 16 Department of Life Sciences John Dalton Building, Faculty of Science and Engineering, Manchester Metropolitan University, Manchester, United Kingdom

**Keywords:** DTI-ALPS index, FMR1 premutation, FXTAS, gene dysregulation, gene expression, glymphatic, neuroinflammation

## Abstract

**Background and objectives:**

Fragile X–associated tremor/ataxia syndrome (FXTAS) is a late-onset neurodegenerative disorder that affects carriers of the *FMR1* premutation (55–200 CGG repeats). It is characterized by motor and cognitive impairments. However, the mechanisms underlying individual susceptibility to FXTAS among carriers remain poorly understood. Emerging evidence suggests that neuroinflammation and glymphatic dysfunction may interact and play key roles in the pathological cascade leading to neurodegeneration. This study aimed to investigate potential glymphatic and/or inflammatory dysfunction in *FMR1* premutation carriers with FXTAS using the diffusion tensor imaging analysis along the perivascular space (DTI-ALPS) index, as well as gene expression and functional enrichment analyses in individuals with FXTAS versus controls.

**Methods:**

We analyzed the DTI-ALPS index in 14 participants with FXTAS and 25 age- and sex-matched controls, and assessed the expression and pathway dysregulation of genes related to neuroinflammation and glymphatic function using Reactome analysis in postmortem brain tissue from 3 individuals with FXTAS and 12 controls and skin fibroblasts from 6 individuals with FXTAS and 3 controls.

**Results:**

The DTI-ALPS index was significantly lower in individuals with FXTAS compared to controls in the right but not left hemisphere (*p* = 0.0051) and globally in both hemispheres (*p* = 0.0473). There was no correlation between lower DTI-ALPS index and increasing CGG repeat length but a trend was observed in males. Reactome analysis revealed downregulation of aquaporin-mediated transport in brain tissue and fibroblasts, upregulation of multiple immune-related and inflammatory pathways, predominantly in brain tissue, and increased circadian-related pathway activity in fibroblasts.

**Discussion:**

Our findings point at glymphatic system dysfunction and neuroinflammation in FXTAS pathophysiology, as evidenced by *in vivo* DTI-ALPS metrics and gene pathway dysregulation and expression in fibroblasts and in postmortem FXTAS brains.

## 1 Introduction

The fragile X messenger ribonucleoprotein 1 (*FMR1*) gene premutation is defined by an expansion of 55–200 CGG trinucleotide repeats in the 5′ untranslated region. Carriers of the premutation are at risk of developing fragile X-associated tremor/ataxia syndrome (FXTAS, OMIM#300623), a late-onset neurodegenerative disorder. Clinically, FXTAS is characterized by intention tremor, gait ataxia, peripheral neuropathy, and parkinsonism, along with cognitive changes that often begin with memory impairment and executive dysfunction and may progress to dementia. The prevalence of FXTAS increases with age; up to 75% of male carriers develop the syndrome, whereas females tend to present with fewer motor symptoms. Neuropathologically, intranuclear inclusions within oligodendrocytes, neurons, and astrocytes are characteristic of FXTAS (Tassone et al., 2023; McLennan et al., 2025). However, mechanisms underlying individual susceptibility to FXTAS remain poorly understood.

FXTAS shares several pathological features with other late-onset neurodegenerative disorders, including protein accumulation, glial dysfunction, and chronic neuroinflammation (Martínez Cerdeño et al., 2018; Dias et al., 2023; Tassone et al., 2023; Zheng et al., 2026). Increasing evidence suggests that glymphatic system health and chronic neuroinflammation may interact to promote a cascade of pathological events leading to impaired waste clearance and subsequent protein aggregation (Cai et al., 2024). In FXTAS, the CGG repeat expansion in the *FMR1* gene results in RNA toxicity, mitochondrial dysfunction, and oxidative stress responses that have been closely linked to neuroinflammatory activation and astrocytic dysfunction (Tassone et al., 2023). Because astrocytes play a central role in glymphatic transport, these pathological alterations may directly contribute to impaired glymphatic clearance mechanisms in FXTAS. In addition, genetic variability in central nervous system (CNS) cell function and immune signaling pathways may further contribute to interindividual variability in protein deposition and neurodegenerative progression (Zhang et al., 2023; Cai et al., 2024). Aging-related epigenetic alterations may exacerbate these processes by promoting maladaptive innate immune responses, including increased basal levels of proinflammatory cytokines, enhanced toll-like receptor (TLR) signaling, inflammasome activation, and reduced anti-inflammatory regulation (Gan et al., 2018). Together, these converging mechanisms may reinforce a cycle of sustained neuroinflammation, protein accumulation, and impaired glymphatic clearance. Framing FXTAS within this interaction between genetic vulnerability, immune dysregulation, and glymphatic dysfunction provides a rationale for investigating glymphatic alterations as a potential contributor to disease pathophysiology.

One genetic factor of particular interest in modulating neurodegenerative susceptibility is the APOe4 allele, which is associated with increased risk of Alzheimer disease. Several studies reported that *FMR1* premutation carriers with FXTAS have a higher frequency of the APOe4 allele compared to both the general population and premutation carriers without FXTAS (Tassone et al., 2023), and APOe4 allele has been associated with a worsening of brain volume and brain degeneration in carriers with no FXTAS symptoms (Jiraanont et al., 2026). At functional level, the APOe4 allele triggers β-amyloid (Aβ) accumulation, and cortical amyloid plaques have been observed in postmortem FXTAS brain tissue (Tassone et al., 2023). Deposited Aβ may further exacerbate pathology by acting as a damage-associated molecular pattern (DAMP), binding to receptors such as TLRs and activating surrounding microglia (Zhang et al., 2023) and neuroinflammation (Cai et al., 2024). Previous neuropathological studies in FXTAS demonstrated increased activation and death of astrocytes, highlighting the importance of glial dysfunction in disease progression (Tassone et al., 2023).

Astrocytes are central part in the glymphatic system, forming perivascular space barriers through their end-foot processes and facilitate solute exchange and clearance between CSF and interstitial fluid via aquaporin-4 (AQP4) channels. Chronic neuroinflammation can disrupt this system by inducing AQP4 depolarization (Cai et al., 2024). Although previous studies investigating the association between AQP4 single nucleotide polymorphisms and FXTAS did not identify significant differences (Elias-Mas et al., 2023), the potential contribution of glymphatic impairment to FXTAS pathology remains a subject of ongoing research.

In recent years, several non-invasive *in vivo* MRI-based techniques aiming to evaluate the glymphatic function were developed. However, no single method emerged as a gold standard, and all currently available approaches present methodological challenges and limitations. Commonly used non-invasive techniques include the assessment of the burden of enlarged perivascular spaces, extent of CSF stagnation, and changes in diffusion parallel to deep medullary veins, namely, the diffusion tensor imaging analysis along the perivascular space (DTI-ALPS) index. The DTI-ALPS index uses diffusion-weighted images to provide a metric that indirectly analyses water diffusivity along perivascular spaces in the cerebral white matter (Taoka et al., 2017). It has been associated to glymphatic function, although it remains the subject of ongoing methodological debate, as it is unclear whether water diffusion in the deep white matter area where it is evaluated can feasibly represent mechanisms that underpin the clearance of large-molecular solutes from the cortex (Eide et al., 2026; Mossige et al., 2026). However, relationships are increasingly reported between the ALPS-index and circadian time and sleep quality (Brendstrup-Brix et al., 2024), cognitive status (Hsiao et al., 2023), CSF biomarker concentrations (Schirge et al., 2025), intrathecal-gadolinium-based contrast agent clearance (Zhang et al., 2021), as well as its sensitivity to cognitive impairment across several neurodegenerative conditions (Costa et al., 2024; Pinho-Correia et al., 2025; Khalafi et al., 2026). One previous study in FXTAS demonstrated an increased burden of enlarged perivascular spaces in the basal ganglia in individuals with FXTAS compared to controls and *FMR1* premutation carriers without FXTAS, suggesting dysfunctional CSF circulation in FXTAS participants (Elias-Mas et al., 2024).

Based on these observations, the present study aimed to investigate the contribution of glymphatic dysfunction and neuroinflammation to the development of FXTAS in *FMR1* premutation carriers. We performed *in vivo* assessment of glymphatic function using the DTI-ALPS index, alongside analyses of gene expression related to glymphatic regulation and neuroinflammation in postmortem brain samples and skin fibroblast cultures from individuals with FXTAS compared to controls. By integrating imaging and molecular approaches, this study seeks to provide a multimodal assessment of mechanisms contributing to FXTAS susceptibility.

## 2 Materials and methods

This is a cross-sectional case–control study integrating neuroimaging and transcriptomic analyses.

### 2.1 Research participants and clinical assessment

All FXTAS individuals were unrelated, recruited from fragile X syndrome families, and met the criteria for either the definite or probable categories of clinical involvement. Considering the broad and sex-dependent clinical spectrum of FXTAS, as well as the recent recognition of the pre-FXTAS category (Liani et al., 2025), only individuals meeting criteria for probable or definite FXTAS were included in the present study. Participants classified as possible FXTAS were excluded to enhance diagnostic accuracy and cohort homogeneity. FXTAS diagnoses were made according to current classification criteria (Hall et al., 2014). Clinical information for the participants with FXTAS, including age, sex, and clinical and radiological diagnostic criteria, is provided in Supplementary Table 1. The control cohort consisted of healthy age- and sex-matched individuals with no clinical evidence of major neurodegenerative disorders. All participants were of Caucasian origin.

### 2.2 MRI acquisitions

For the DTI-ALPS study, 39 participants were included: 14 *FMR1* premutation carriers with FXTAS (7 males vs. 7 females) and 25 controls (12 males vs. 13 females). MRI acquisitions were performed in two different centers: images of 11 individuals with FXTAS (4 probable and 7 definite) and 8 controls were acquired on a 3 T MRI Philips Achieva at Hospital de la Santa Creu i Sant Pau between 2000 and 2013; and the remaining participants—3 individuals with FXTAS (1 probable and 2 definite) and 17 controls—were acquired on a 3 T Siemens MAGNETOM Prisma at Hospital Clínic Barcelona between 2023 and 2025.

MRI data of 19 subjects were collected using a 3 T MR scanner (Philips Achieva) with 16 channel orthogonal head coil, including the following sequences: 3D T1-weighted, T2 fluid-attenuated inversion recovery (T2-FLAIR) and diffusion weighted MRI (DWI). T1-weighted image was acquired using a 3D-MPRAGE Turbo Field Echo covering the whole brain, repetition time (TR) = 6.7 ms, echo time (TE) = 3.1 ms, flip angle = 8°, slice thickness = 1.2 with no gap between slices, field of view = 256 × 256 mm^2^, voxel size 1.2 × 0.889 × 0.889 mm^3^. T2-FLAIR was acquired with TR = 8,000 ms, TE = 356 ms, inversion time = 2,400 ms, field of view = 256 × 256 mm^2^, voxel size = 0.399 × 0.399 × 0.5 mm^3^. DWI were acquired using a SENSE single shot EPI sequence with TR = 8,132 ms, TE = 60 ms, field of view = 224 × 224 mm^2^, voxel size = 1.75 × 1.75 × 2 mm^3^, including 15 gradient directions with *b*-value = 800 s/mm^2^, and one baseline image without diffusion weighting (*b*-value = 0 s/mm^2^).

MRI data from 20 participants were collected on a 3 T MR Scanner (Siemens MAGNETOM Prisma) with a 32-channel head coil, including the following sequences: 3D T1-weighted image, 3D T2w-FLAIR and DWI. T1-weighted image was acquired with a 3D-MPRAGE sequence covering the whole brain, TR = 2,500 ms, TE = 4.37 ms, flip angle = 7°, field of view = 256 × 256 mm^2^, voxel size = 1 × 1 × 1 mm^3^; T2-FLAIR was acquired with TR = 5,000 ms, TE = 388 ms, field of view = 256 × 256 mm^2^, inversion time = 1,800 ms, slice thickness = 1 mm with no gap between slices, DWI was acquired with a multi-shell protocol including 6, 64 and 64 gradient directions with *b*-values = 500 s/mm^2^, 1,000 s/mm^2^ and 2,000 s/mm^2^, respectively, and 14 baseline images without diffusion weighting, TR = 3,000 ms, TE = 113 ms, field of view = 224 × 224 mm^2^, voxel size = 2 × 2 × 2 mm^3^. An extra baseline image with the same parameters was acquired with inverse phase direction to further correct for field distortions.

All MR images were anonymized for further analysis.

### 2.3 Diffusion tensor image analysis along the perivascular space (DTI-ALPS)

Diffusion-weighted images were processed to compute the DTI-ALPS, including correction for eddy currents and EPI distortions (using FSL top-up in the Siemens cohort and elastic registration to the anatomical T1-weighted image using ANTs in the Philips acquisitions) and estimation of the DTI using dipy (Garyfallidis et al., 2014). DTI ALPS was computed from the diffusion tensor components as defined in Taoka et al. (2017):

ALPS index=(mean(Dxproj,Dxassoc))/(mean(Dyproj,Dzassoc))

where Dx, Dy and Dz are the diagonal elements of the diffusion tensor in areas identified as projection fibers and association fibers. These areas were manually defined using ITK-Snap (Yushkevich et al., 2006) on the color-fractional anisotropy map estimated from the DTI by one experienced rater. Five mm diameter regions were drawn on the projections and association fibers as described in Taoka et al. (2017). For the multi-shell acquisition only *b*-value = 1,000 s/mm^2^ shell was used for DTI estimation to have the most similar parameters to the one-shell acquisition and limit the differences associated within the acquisition protocol.

### 2.4 Gene expression and functional enrichment analysis

RNA sequencing data and functional enrichment analysis were obtained from previous studies from our group, including skin fibroblasts and postmortem prefrontal cortex brain samples (Alvarez-Mora et al., 2023). RNA sequencing was performed on skin fibroblasts from 6 individuals with FXTAS and 3 controls, as well as on postmortem prefrontal cortex samples from 3 *FMR1* premutation carriers with FXTAS and 12 control individuals. Differential gene expression analysis was performed as previously described (Alvarez-Mora et al., 2023).

Functional enrichment analysis of the differential expression profiles between patients and controls was conducted using the Gene Set Analysis (GSA) method implemented in the mdgsa R package with Gene Ontology (GO) and Reactome databases as functional annotations (Montaner and Dopazo, 2010). This approach identifies significantly up- or down-regulated groups of functionally related genes from lists ranked according to differential gene expression. Because multiple functional terms are tested simultaneously, *p*-values were adjusted using the Benjamini–Hochberg method (Benjamini and Hochberg, 1995), and pathways with an adjusted *p*-value < 0.05 were considered significantly deregulated. The GSA method is based on a logistic regression model in which significant positive log odds ratios (LOR) indicate up-regulated pathways, whereas significant negative LOR indicate down-regulated pathways.

### 2.5 Statistics

Results were expressed as mean ± standard deviation (SD). For descriptive analyses, a chi-square test was used to assess differences in gender between groups, and the non-parametric Mann–Whitney U test was used to assess differences in age between groups. The non-parametric test was used due to the non-normality of the distribution. For DTI-ALPS analysis, an ANCOVA was conducted, with scanner included as a covariate. A partial Spearman correlation was performed between the ALPS index and the number of CGG repeats, controlling for scanner type, using jamovi version 2.6.26.^1^ A *p*-value < 0.05 was considered statistically significant.

## 3 Results

### 3.1 Participant characteristics

A total of 14 individuals with FXTAS (5 probable and 9 definite) and 25 sex- and age-matched healthy controls were included in the DTI-ALPS index analysis. There were no statistically significant differences between the two groups in terms of age (*p* = 0.284) or sex distribution (*p* = 0.905). Table 1 summarizes the clinical and molecular characteristics of the DTI-ALPS study the participants.

**Table 1:** Clinical and molecular characteristics of the DTI-ALPS study participants.

DTI-ALPS study	FXTAS (*n* = 14)	Control (*n* = 25)	*p-*value
Age at study participation			
Mean (±SD)	67.1 ± 11.0	64.8 ± 9.34	0.284
Sex			
Female (%)	7 (50%)	13 (52%)	0.905
Males (%)	7 (50%)	12 (48%)	
Clinical diagnosis			
Definite	9	Not applicable	Not applicable
Probable	5	Not applicable	Not applicable
Age at FXTAS diagnosis			
Mean (±SD)	63.7 ± 9.6	Not applicable	Not applicable
*FMR1* CGG repeat size			
Mean ± SD [Range]	88.4 ± 28.2 [60–156]	29.8 ± 1.97 [26–34.5]	<0.001

### 3.2 DTI-ALPS findings

**Figure 1:**
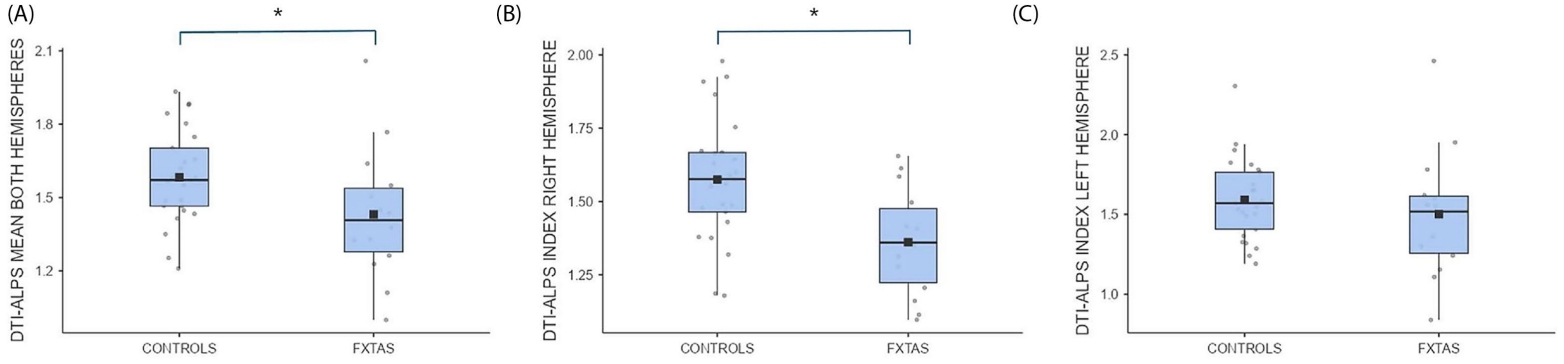
Box plots showing the distribution of the DTI-ALPS index in the mean both hemispheres **(A)**, right hemisphere **(B)**, and left hemisphere **(C)** in FXTAS participants versus controls. DTI-ALPS: Diffusion tensor image analysis along the perivascular space. Error bars represent the standard error. *p < 0.05.

Table 2 shows the mean DTI-ALPS index (±SD) and range in controls and FXTAS participants. While no differences in the DTI-ALPS index were found when analyzing the data from the two scanners separately, analysis of the combined dataset showed that the mean index across both hemispheres was lower in FXTAS, with a significant difference observed in the right hemisphere (*p* = 0.0051) and for the average across both hemispheres (*p* = 0.0473) (Figure 1).

**Table 2:** Mean DTI-ALPS index (±SD) and range in controls and FXTAS participants.

Hemisphere	Controls Mean (± SD) [Range]	FXTAS Mean (± SD) [Range]	ANCOVA *p*-value
Both hemispheres (mean)	1.58 (±0.19) [1.21–1.93]	1.43 (±0.27) [1.00–2.06]	**0.0473**
Right hemisphere	1.57 (±0.21) [1.18–1.98]	1.36 (±0.18) [1.10–1.66]	**0.0051**
Left hemisphere	1.59 (±0.25) [1.19–2.30]	1.50 (±0.40) [0.84–2.46]	0.3164

Bold, significant at *p* < 0.05.

### 3.3 DTI-ALPS index – *FMR1* CGG repeat size correlation

**Figure 2:**
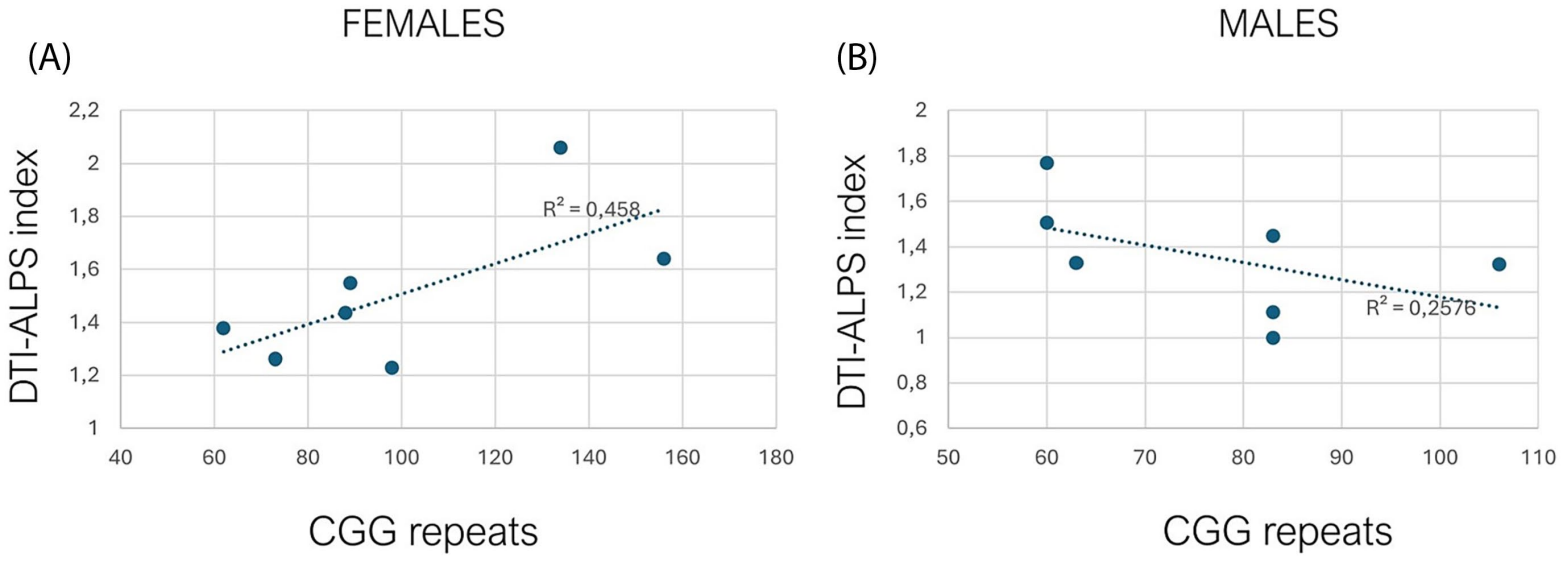
Scatter plots with fitted linear regression lines illustrating the relationship between CGG repeat length and the DTI-ALPS index in the mean of both hemispheres in **(A)** females and **(B)** males.

No significant relationships were found between DTI-ALPS index in the right hemisphere and CGG (*p* = 0.801), DTI-ALPS index in the left hemisphere and CGG (*p* = 0.753), or DTI-ALPS index mean both hemispheres and CGG (*p* = 0.790) in FXTAS group. When analyzed separately by sex to avoid the confounding effect of X-chromosome inactivation in females, no statistically significant associations were observed between the mean bilateral ALPS index and CGG repeat size in either males (*p* = 0.106) or females (*p* = 0.163). However, a trend toward lower DTI-ALPS index values with increasing CGG repeat length was noted in males (Figure 2).

### 3.4 Gene pathway analysis in brain and fibroblasts samples

Functional enrichment analysis using the Reactome database revealed both shared and tissue-specific pathway alterations in FXTAS brain and fibroblast samples (Table 3). Pathways related to aquaporin-mediated transport were significantly downregulated in both prefrontal cortex and skin fibroblasts, with negative logarithm of the odds ratio (LOR) observed in both tissues, indicating a consistent decrease in the expression of genes involved in water transport.

**Table 3:** Neuroinflammation and glymphatic system-related Reactome pathways and Gene Ontology (GO) enrichment analysis deregulated in individuals with FXTAS compared to controls.

PATH_ID	PATH_NAME	Brain samples			Fibroblast sample		
		Size	*p*_adj_	LOR	Size	*p*_adj_	LOR
GO:0006954	inflammatory response	242	**1.70E−13**	0.6037	252	**0.000006432**	0.3535
R-HSA-198933	Immunoregulatory interactions between a Lymphoid and a non-Lymphoid cell	71	**1.32E−07**	0.9048	52	0.321	−0.1922
R-HSA-5260271	Diseases of Immune System	24	**0.000001481**	1.166	22	0.1749	0.3964
R-HSA-5602358	Diseases associated with the TLR signaling cascade	24	**0.000001481**	1.166	22	0.1749	0.3964
R-HSA-6783783	Interleukin-10 signaling	23	**0.00001095**	0.9955	33	**0.00009264**	0.8284
R-HSA-6785807	Interleukin-4 and Interleukin-13 signaling	72	**0.00003701**	0.5708	85	**0.01395**	0.3503
R-HSA-449147	Signaling by Interleukins	343	**0.00006796**	0.2573	362	**0.003564**	0.194
R-HSA-5603041	IRAK4 deficiency (TLR2/4)	11	**0.0003237**	1.316	10	0.708	0.1889
R-HSA-5686938	Regulation of TLR by endogenous ligand	12	**0.0005556**	1.099	11	0.3286	0.4159
R-HSA-5602498	MyD88 deficiency (TLR2/4)	10	**0.001426**	1.234	No matching		
R-HSA-1679131	Trafficking and processing of endosomal TLR	11	**0.02304**	0.899	11	0.5221	−0.2846
R-HSA-6783589	Interleukin-6 family signaling	19	**0.03658**	0.6224	22	**0.02921**	0.613
R-HSA-445717	Aquaporin-mediated transport	38	**0.0428**	−0.4453	34	**0.02403**	−0.5161
R-HSA-8983432	Interleukin-15 signaling	11	**0.04681**	0.7734	13	0.6702	−0.1852
R-HSA-400253	Circadian Clock	64	0.1944	−0.2324	65	**0.002011**	0.4825
GO:0032922	circadian regulation of gene expression	58	0.4739	−0.1573	58	**0.04006**	0.4174
GO:0007623	Circadian rhythm	43	0.8424	−0.06728	45	**0.002744**	0.6643
GO:0043153	entrainment of circadian clock by photoperiod	24	0.9221	0.04407	25	**0.03007**	0.6451

LOR, logarithm of the odds ratio. Bold, significant at *p*_adj_ < 0.05.

Circadian-related pathways showed a tissue-specific pattern of deregulation. In brain samples, no significant enrichment was detected, whereas multiple circadian-related pathways were significantly enriched in fibroblast samples, all showing positive LOR, indicating upregulation of circadian-associated genes in skin fibroblast cultures from FXTAS individuals.

Immune- and inflammation-related pathways were prominently enriched in both tissues, with stronger statistical significance and higher effect sizes observed in brain samples. In the prefrontal cortex, several interleukin-associated pathways were significantly enriched. Pathways related to immunoregulatory interactions between lymphoid and non-lymphoid cells, diseases of the immune system, and multiple TLR-associated pathways were also significantly enriched in brain tissue. In fibroblast samples, enrichment of interleukin-related pathways and the inflammatory response was also observed; however, fewer immune-related pathways reached statistical significance, and effect sizes were generally lower compared with brain samples. Notably, TLR-related pathways did not reach statistical significance in fibroblasts. When analyzing expression levels of genes related to these pathways, 7 TLR genes were identified as significantly deregulated in FXTAS brain samples (Table 4). None of the skin fibroblast samples from FXTAS patients reached a significant *p*-Value.

**Table 4:** Neuroinflammation-related genes significantly deregulated in postmortem brain and fibroblasts samples of individuals with FXTAS compared to controls.

Brain samples			Fibroblast samples	
Gene	Fold change	*p*_adj_	Fold change	*p*_adj_
*TLR1*	1.683	**0.001692**	1.804	0.5021
*TLR6*	1.533	**0.001935**	0.5011	0.9194
*TLR5*	1.718	**0.01145**	0.6759	0.9923
*TLR7*	1.932	**0.01374**		
*TLR10*	1.741	**0.01963**		
*TLR3*	1.121	**0.02689**		
*TLR2*	1.897	**0.03779**	0.9924	0.9453

Bold, significant at *p*_adj_ < 0.05.

## 4 Discussion

In this study, we integrated diffusion MRI–based assessment of glymphatic function with transcriptomic pathway analysis to investigate convergent mechanisms underlying FXTAS pathology. Our findings provide some evidence of impaired glymphatic system in FXTAS, as measured by DTI-ALPS, accompanied by molecular alterations involving aquaporin-mediated transport, immune signaling, inflammatory pathways and circadian regulation. Together, these results suggest that disruptions in fluid homeostasis and neuroinflammation may contribute to FXTAS pathophysiology.

### 4.1 Glymphatic function

Using the DTI-ALPS index as an indirect marker of glymphatic function, we observed significantly reduced values in individuals with FXTAS compared with healthy controls, affecting the right hemisphere. Because the DTI-ALPS index reflects the Brownian motion of water molecules in the radial direction at the level of the lateral ventricular body, its reduction is suggestive of impaired perivenular efflux of interstitial fluid in the deep white matter. The lateralized pattern observed here aligns with prior neuroimaging studies and suggests that glymphatic dysfunction may not be uniformly distributed across the brain (Steward et al., 2021; Ocklenburg et al., 2024; Liu et al., 2025; Miao et al., 2025). Similar reductions in the DTI-ALPS index have been reported in other neurodegenerative disorders, including Alzheimer’s disease, Parkinson’s disease, and Huntington’s disease, where lower values are associated with cognitive and motor impairment, abnormal white matter signal, enlarged perivascular spaces, brain atrophy, and increased amyloid and tau deposition (Taoka et al., 2024; Yin et al., 2025). These further supports a broader role for glymphatic dysfunction in neurodegeneration including FXTAS. The fact that the brain tissue used for postmortem transcriptomic analyses was derived from the prefrontal cortex, whereas DTI-ALPS analysis reflects changes in the deep white matter, may provide converging evidence of glymphatic system dysfunction across both cortical and deep white matter regions. However, it should be noted that evidence supporting the presence and function of the glymphatic system in white matter remains limited.

There was no correlation between DTI-ALPS and *FMR1* CGG repeat length. However, this finding should be interpreted cautiously given the small sample size. A trend toward lower glymphatic activity with increasing CGG repeat size was observed in males with FXTAS. This pattern is consistent with prior reports showing that mid-range and higher CGG repeat expansions within the premutation range are associated with increased cognitive and motor symptom burden (Jacquemont et al., 2007). In contrast, no comparable trend was observed in females. However, the absence of data on X-chromosome inactivation status in brain tissue limits interpretation of sex-specific effects and precludes definitive conclusions regarding the relationship between CGG repeat length and glymphatic function in females.

At the molecular level, aquaporin-mediated transport pathways showed a modest but statistically significant downregulation in FXTAS postmortem brain tissue and showed a consistent reduction in fibroblasts, providing a potential mechanistic link to the observed glymphatic impairment. However, these findings should be interpreted with caution, as the MRI-derived measures of glymphatic function and the molecular analyses were obtained from independent patient cohorts. Aquaporins are membrane water channels that facilitate bidirectional water transport, and in the central nervous system, AQP4 is highly enriched in astrocytic end feet surrounding perivascular spaces, where it plays a critical role in cerebrospinal fluid–interstitial fluid exchange and glymphatic clearance. Experimental deletion of AQP4 in mice slows perivascular exchange, impairs amyloid-β clearance, and promotes plaque formation, while human neuropathological studies have demonstrated altered AQP4 expression with aging and in association with α-synuclein deposition in Parkinson’s disease (Hoshi et al., 2017; Zeppenfeld et al., 2017). Loss of perivascular AQP4 polarization in Alzheimer’s disease was linked to increased amyloid-β and tau pathology and to cognitive decline preceding dementia onset. Taken together, these findings raise the possibility that dysregulation of aquaporin-mediated transport could be associated with impaired glymphatic clearance in FXTAS, facilitating protein accumulation and neurodegeneration.

Circadian-related pathways showed modest significant enrichment in fibroblasts but not in postmortem brain samples. This divergence could potentially reflect both biological and technical factors. Peripheral fibroblasts retain robust, cell-autonomous circadian clocks and are less susceptible to confounders such as time of death and postmortem interval, which can attenuate rhythmic gene expression in brain tissue. The upregulation of circadian pathways in fibroblasts may therefore reflect preserved or compensatory circadian responses to cellular stress in FXTAS and underscores the value of peripheral models for capturing disease-relevant molecular signatures that may be masked in postmortem brain analyses.

### 4.2 Immune and inflammatory pathways

Immune and inflammatory pathways emerged as another prominent feature of FXTAS, with a markedly stronger signature in prefrontal cortex tissue compared with fibroblasts. Brain samples showed significant enrichment of multiple interleukin signaling pathways and TLR-associated pathways, consistent with a pronounced neuroinflammatory state and aligning with growing evidence implicating innate immune activation and microglial dysfunction in FXTAS (Martínez Cerdeño et al., 2018; Dufour et al., 2021; Dufour et al., 2024). TLRs are pattern-recognition receptors that respond to endogenous and exogenous ligands and promote microglial activation through downstream signaling cascades, including NF-κB activation via the adaptor protein MyD88 (Gao et al., 2023). Interleukin-1 receptor–associated kinase 4 (IRAK-4), a key downstream component of both TLR and interleukin-1 receptor signaling, has been shown to exhibit increased expression and activity in Alzheimer’s disease (Gazestani et al., 2023). Consistent with this, microglia overexpressing neuroinflammatory genes expand as Alzheimer’s disease pathology progresses, and postmortem transcriptomic studies in Parkinson’s disease reveal stage-specific enrichment of immune and inflammatory pathways (Hoozemans et al., 2014; Cappelletti et al., 2023; Gazestani et al., 2023). Furthermore, a previous study in FXTAS postmortem cerebellar tissue found significant elevations in the cytokines, interleukin-12 and TNF-α (Dufour et al., 2021). Together, these observations support a model in which activated microglia play a central role in FXTAS progression, potentially contributing both to clearance of pathological protein aggregates and to neuronal injury via sustained release of pro-inflammatory mediators. In contrast, although fibroblasts exhibited enrichment of some immune-related pathways, TLR-associated pathways did not reach statistical significance, suggesting that full activation of inflammatory cascades may depend on central nervous system–specific cellular interactions absent in peripheral cells.

### 4.3 Limitations

Several limitations should be acknowledged. The DTI-ALPS index provides an indirect and still debated measure of glymphatic function in the deep white matter and cannot capture dynamic fluid flow in the whole brain especially the cerebral cortex where the glymphatic system was first reported; nevertheless, it is widely used, correlates with the gold standard measure of glymphatic system using intrathecal contrast-enhanced MRI (Zhang et al., 2021; Boyd et al., 2024), and no non-invasive method has yet been shown to more accurately assess glymphatic function in humans. MRI data were acquired using two 3 T scanners with differing acquisition parameters, although these effects were accounted for statistically. The small sample size, particularly for postmortem transcriptomic analyses, limits statistical power and generalizability but is expected given the rarity of FXTAS. Additionally, pathway enrichment analyses rely on non–tissue-specific curated databases, which may introduce bias. Finally, although the integration of molecular and imaging data represents a strength of this study, these measures were obtained from independent cohorts, limiting the ability to establish direct or causal relationships between MRI-derived metrics and molecular findings.

### 4.4 Conclusion

In summary, our findings support a model in which FXTAS is characterized by impaired glymphatic function, potentially linked to aquaporin dysregulation, together with neuroinflammatory and altered immune-related pathways. These results identify glymphatic dysfunction and neuroinflammation as a potential feature of FXTAS and highlight the value of integrated imaging–molecular approaches for advancing mechanistic understanding and identifying potential therapeutic targets.

## Data Availability

The raw data supporting the conclusions of this article will be made available by the authors, without undue reservation.

## Footnotes

1
www.jamovi.org
