# Supplementary material for: Glymphatic dysfunction and neuroinflammation in FXTAS: evidence from DTI-ALPS and gene expression analysis
Source: Front Mol Neurosci. 2026 May 28;19:1856101. doi: 10.3389/fnmol.2026.1856101 (PMC13280973; doi:10.3389/fnmol.2026.1856101)
Supplement: Supplementary file 1 [file Table_1.DOCX]

Supplementary table 1. Clinical information for the FXTAS participants

| SEX | Age at study participation | Clinical diagnostic criteria | Radiological diagnostic criteria | FXTAS diagnostic criteria |
| --- | --- | --- | --- | --- |
| FEMALE | 75 | 2 major | 1 major + 2 minor | DEFINITE |
| FEMALE | 77 | 2 major | 1 major | DEFINITE |
| FEMALE | 56 | 1 major | 1 major | DEFINTE |
| FEMALE | 42 | 1 major | 1major + 1 minor | DEFINITE |
| FEMALE | 73 | 2 major + 1 minor | 1 major | DEFINITE |
| MALE | 77 | 2 major | 1 major + 1 minor | DEFINITE |
| MALE | 70 | 1 major | 2 major | DEFINITE |
| MALE | 65 | 2 major | 1 major + 1 minor | DEFINITE |
| MALE | 79 | 2 major + 1 minor | 2 major + 2 minor | DEFINITE |
| FEMALE | 51 | 1 minor | 1 major + 1 minor | PROBABLE |
| FEMALE | 73 | 2 majors | 0 | PROBABLE |
| MALE | 60 | 1 minor | 1 major + 1 minor | PROBABLE |
| MALE | 71 | 2 major + 1 minor | 1 minor | PROBABLE |
| MALE | 70 | 2 major + 1 minor | 2 minor | PROBABLE |
